# Sentinels tracking viruses in various ecosystems: Towards a One Health approach

**DOI:** 10.1371/journal.ppat.1013141

**Published:** 2025-05-28

**Authors:** Léo Blondet, Amour Mouanda Sounda, Matthieu Fritz, Denis Filloux, Michel Yvon, Stéphane Blanc, Arvind Varsani, Roch Fabien Niama, Eric M. Leroy, Philippe Roumagnac

**Affiliations:** 1 CIRAD, UMR PHIM, Montpellier, France; 2 PHIM Plant Health Institute, Univ Montpellier, CIRAD, INRAE, Institut Agro, IRD, Montpellier, France; 3 Université Marien Ngouabi, Brazzaville, Republic of the Congo; 4 UMR TransVIHMI, Université de Montpellier, IRD, Inserm, Montpellier, France; 5 Laboratoire National de Santé Publique, Brazzaville, Republic of the Congo; 6 The Biodesign Center for Fundamental and Applied Microbiomics, Center for Evolution and Medicine, School of Life Sciences, Arizona State University, Tempe, Arizona, United States of America; 7 Structural Biology Research Unit, Department of Integrative Biomedical Sciences, University of Cape Town, Observatory, Cape Town, South Africa; Shanghai Center for Plant Stress Biology, CHINA

## The urgent need for innovative sampling strategies to reveal an ever-elusive viral diversity

Viruses are widely regarded as the most abundant and genetically diverse biological entity on Earth, with multiple studies converging towards an estimated number of viral particles present globally on the order of 10^31^ [1,2]. They infect organisms in all domains of life and are essential for ecosystem health, resilience, and function [3]. Despite their prevalence, only a fraction of viral species is known and classified by the International Committee on Taxonomy of Viruses (ICTV, 14690 species as of April 2024 [4]). Most of the known viruses are biased towards those infecting humans, livestock, and crops, reflecting largely human-centric approach to virus research. The growing number of outbreaks in past decades caused by animal or plant viruses such as Ebola virus, mpox, SARS-CoV-2, influenza virus, or tomato yellow leaf curl virus, underscores the gaps in our understanding of viral ecology [5,6]. This lack of knowledge limits our comprehension of the patterns and processes behind viral evolution, which hinders the early detection and monitoring of emerging pathogens, having dramatic consequences on human, animal and plant health, and hence, food safety. It is clear that ecosystem-level knowledge is crucial for improving global health, a gap that the One Health strategy aims to address by integrating medical, social, environmental, and veterinary sciences [3,7]. The One Health approach recognises that all components of an ecosystem are interconnected and can directly or indirectly affect one another. This notion has been reinforced by the advancement of viral metagenomics, allowing the identification of both known and novel viruses in a very short time [8,9]. Viral metagenomics involves sequencing the nucleic acid content of viruses within samples without prior knowledge of the viruses present. It has been applied to multiple types of samples (*i.e.*, environmental, whole individual, gut) and in various fields such as ecology, diagnostics, monitoring and surveillance. Despite the decreasing cost of sequencing, the challenge of exhaustively sampling all the viruses hosted by species across entire ecosystems remains extremely difficult, if not impossible. Many species are particularly hard to find and capture due to the ecosystems they inhabit and their unique ecological characteristics. Additionally, national, and international regulations prohibit the sampling of many wild species, limiting the amount of available biological data. In this case, non-invasive sampling from faeces can be utilised, though it only offers a partial view of the viral community. Consequently, there is a pressing need to develop new sampling approaches. Here, we aim to argue for the potential of using sentinels—species that respond quickly, measurably, and in a timely manner to environmental changes—as new large-scale and thorough sampling strategies combined with molecular tools to efficiently document the virosphere and monitor disease emergence.

## Animal and plant sentinels: Virus collectors to be chosen with precision

**Fig 1:**
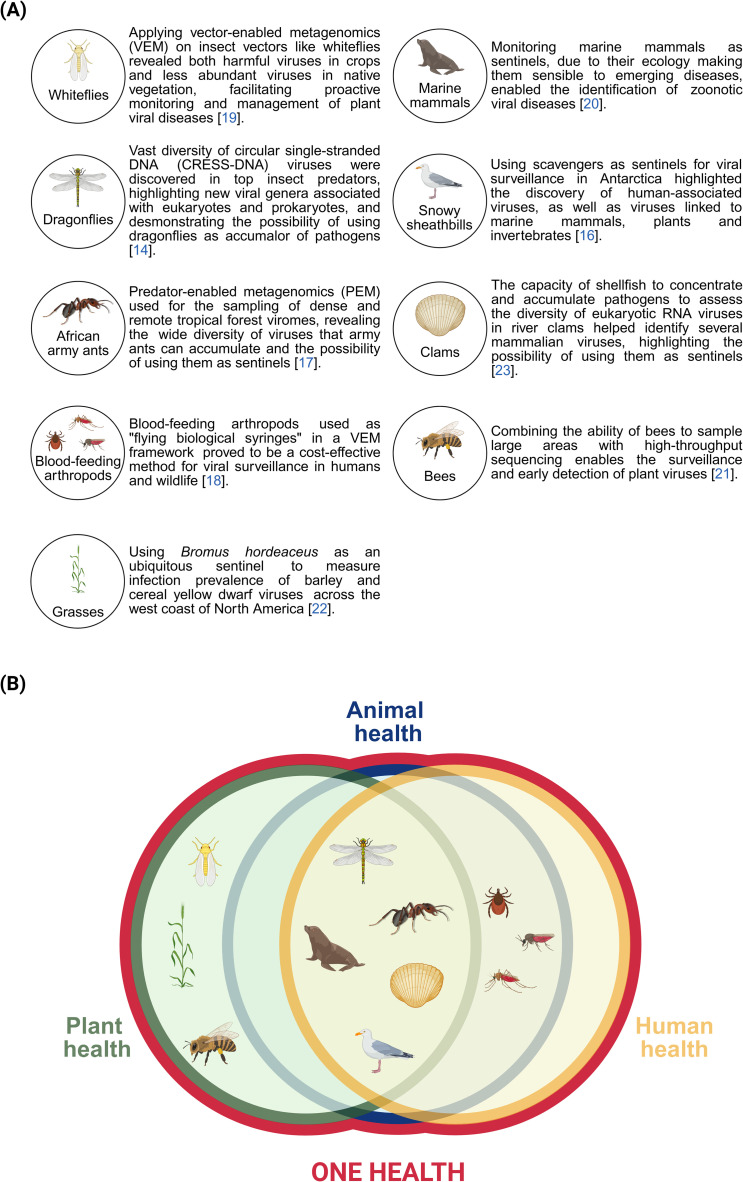
The use of sentinels as viral detectors (created with Biorender). **(A)** Non-exhaustive list of species used for viral detections and the findings linked to them. **(B)** Implementation of sentinels within one of the three components of the One Health strategy.

As anthropogenic activities accelerate climate change, predicting how ecosystems will respond becomes increasingly challenging. Sentinels have been proposed as valuable tools for monitoring and protecting socio-ecological systems [10]. A classic example of a sentinel species is the Canary bird, used by coal miners to provide early warnings of toxic carbon monoxide emissions [11]. While sentinels can be used to detect hazardous chemicals and pollutants, they can also serve as indicators of biodiversity, biotic and abiotic changes, or to detect the presence of viruses [10]. Different sentinels have been extremely helpful in detecting changes in the virosphere across diverse ecological and epidemiological contexts, offering early warnings of potential outbreaks (Fig 1A) [12].

**Fig 2:**
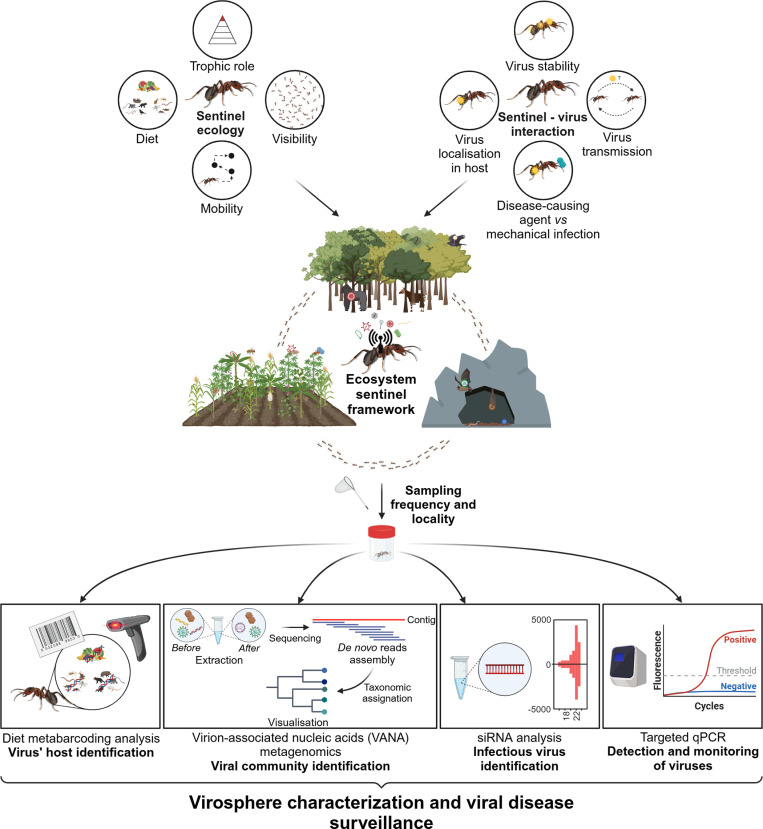
Designing an ecosystem sentinel framework for viral surveillance using army ants (created with Biorender). To characterise the virosphere and monitor specific viral diseases within a particular context, an ecosystem and sentinel species are first identified to establish the ecosystem sentinel framework. The selection of species is based on its ecology and interactions with the targeted viruses to improve detection. A sampling strategy is then designed, and various molecular techniques are used to analyse the sentinel virome.

To be most effective, a sentinel must possess specific ecological traits and interact with viruses in a way that improves the cost- and time-effectiveness of monitoring. Within a specific ecosystem, these two elements form the ecosystem sentinel framework and should be integrated in one or more components of the One Health strategy [12] (Figs 1B and 2). Sentinels should inhabit the same environment as the hosts and viruses being targeted, possess the capacity to carry, accumulate, and/or become infected by the viruses, and allow at least some of their genetic material to remain stable long enough for detection. Additional ecological attributes can enhance a sentinel’s utility for viral surveillance. The intensity of trophic interactions, which refers to a species’ connection to other ecosystem components, can serve as a predictive criterion for identifying effective sentinels [13]. Top predators and scavengers are particularly effective due to their strong connections with their resources and their exposure to a variety of prey, making them more likely to accumulate a broad range of pathogens [14–17]. Pathogen vectors are also strong candidates for sentinel surveillance because they play a critical role in transmitting pathogens [18,19]. Sentinels must also be easily sampled and observable for timely monitoring. Species with broad geographic ranges and those with specific ecological niches are particularly valuable because they can provide data from diverse locations and ecosystems, or from more specialised environments, respectively [16,17,20–22]. Species that feed through filtration are also important, as they can concentrate viruses from their environment [23]. Species serving as reservoirs, alternate or secondary hosts for specific viruses have also proven valuable in addressing specialised questions related to viral virulence or host immunity [24]. However, this focus somewhat deviates from the scope proposed in this review. Overall, an effective ecosystem sentinel framework for virus detection and surveillance takes into account both the sentinel’s ecology and its interactions with viruses within the ecosystem. In this regard, applying viral metagenomics to a sentinel does provide valuable insights into the virosphere, and generalising such approaches would without a doubt improve early detection and potentiate surveillance efforts (Fig 2).

## Sentinels in action for viral monitoring in hard-to-reach environments

Several pioneering studies have demonstrated that top-end insects or feline predators can be useful indicators of viruses in the phylum *Cressdnaviricota* in terrestrial ecosystems [14,15,25]. While these studies were noteworthy for demonstrating proof-of-concept of sentinel framework, they were limited in scope as they focussed solely on one particular phylum of viruses. More recently, two other studies have gone a step further by characterising the virome (*i.e*., the overall collection of viral sequences) of animal sentinels: one conducted in a remote tropical rainforest in Gabon using army ants of the genus *Dorylus* [17], the other carried out in the Antarctic Peninsula and South Shetland using snowy sheathbill (*Chionis albus*) [16]. Both species inhabit ecosystems characterised by their remoteness and extreme meteorological conditions, making these environments hostile and hard-to-reach by surveillance scientists. Despite tropical forested regions being hotspots for emerging diseases and Antarctica a region for putative endemic viral evolution, little is known about the viruses circulating in these regions [26,27]. The nomadic behaviour of army ants (*i.e.,* frequently relocating their nests) and migratory behaviour of snowy sheathbills (*i.e*., travelling between Graham Land in Antarctica and the Río de la Plata) make them well-suited to serve as sentinels for viral diversity in their ecosystems as they are exposed to a wide range of biotic and abiotic conditions [28,29]. Hence, both species have a wide diet, including invertebrates, live or dead vertebrates, and photosynthetic eukaryotes [28–30]. By analysing the virome of army ants and snowy sheathbills, 56 and 17 viral families were identified, respectively [16,17]. Interestingly, these two studies highlighted the discovery of sequences with high identity with that of human viruses and a cyclovirus in army ants, a Sapovirus GII and a gammaherpesvirus in snowy sheathbills, but also with plant viruses. These findings provide insight into the complex viral networks that may exist between humans, animals (*e.g*., birds and invertebrates) and plants, highlighting the potential for conceptualising *ad hoc* sentinel frameworks for viral monitoring and surveillance of difficult-to-access ecosystems.

## Sentinel-based epidemiological surveillance: An approach that still needs validation

These studies have raised several intriguing and unresolved questions that are crucial for determining the best approach to enhance inferences made from sentinels. While sentinels appear to sample/accumulate a significant variety of viral genomic sequences, it is essential to determine whether the viral diversity they accumulate accurately reflects the one circulating within the ecosystems they inhabit. It must also be established whether this representativeness remains consistent across ecosystems and over time through longitudinal sampling at various locations. Furthermore, understanding where viral nucleotide sequences localise within a sentinel and how long they persist on or inside them would provide valuable insights into the interaction between sentinels and viruses, potentially leading to more efficient sampling strategies. Evaluating the variation of viral diversity between individuals coming from, in the specific case of ants, different castes (workers, soldiers, queens, and males), as well as colonies, species and ecological niches would also help optimise the sampling scheme. Once an ecosystem sentinel framework is established, viral metagenomics techniques need to be combined with complementary molecular methods to evaluate the reliability of sentinels (Fig 2). Specifically, identifying the hosts through which sentinels accumulate viral sequences, using diet metabarcoding analyses, could help pinpoint potential reservoirs and locations where specific viruses are present, facilitating the implementation of mitigation measures. It is also important to determine whether viruses are infectious (*i.e.,* replicate within sentinels) or in a transitory phase in sentinels, using small interfering RNAs (siRNA) for instance, which are produced as host antiviral RNA interference (RNAi) response [31]. Viruses with potential risk of spillover should be detected and monitored using targeted quantitative polymerase chain reaction (qPCR). Tracking sentinels foraging areas using biologging technology [32] could also provide important insights into their foraging behaviour and offer a clearer understanding of the environments in which they develop their virome. Even after addressing these questions, we must still determine how to assess the risk of any spillover into other species based on the viral sequences identified in a sentinel’s virome. While we use army ants and snowy sheathbills as examples of sentinel frameworks, other species have shown their potential in different contexts (Fig 1A). Thus, it is now essential to evaluate whether different species, such as termites with their xylophagous feeding habits [33] or spiders as top insect predators [34], whose virome has been documented but not within a sentinel framework, could be good candidates for use as sentinels of specific ecosystems. Developing these studies should be the first step towards establishing effective ecosystem sentinel frameworks. Guided by a One Health perspective, they could play a pivotal role in preventing future outbreaks.
